# A Short-Chain Fatty Acid, Butyrate, Suppresses the Hyperexcitability of Rat Nociceptive Primary Neurons Involved in Inflammatory Hyperalgesia

**DOI:** 10.3390/molecules30112407

**Published:** 2025-05-30

**Authors:** Yukito Sashide, Syogo Utugi, Mamoru Takeda

**Affiliations:** Laboratory of Food and Physiological Sciences, Department of Life and Food Sciences, School of Life and Environmental Sciences, Azabu University, 1-17-71 Fuchinobe, Chuo-ku, Sagamihara 252-5201, Kanagawa, Japan; sashide012@hotmail.co.jp (Y.S.); f22015@azabu-u.ac.jp (S.U.)

**Keywords:** extracellular single-unit recording, gut microbiota, SCFAs, inflammation, peripheral sensitization, COX-2, butyrate

## Abstract

While gut microbiota-derived short-chain fatty acids (SCFAs) are recognized to modulate pathological pain by decreasing inflammation, the neurophysiological basis for SCFAs, butyrate’s ability to reduce hyperexcitability in nociceptive primary neurons during inflammatory conditions is still unknown. The objective of this study was to determine, using in vivo conditions, whether systemic butyrate administration attenuates inflammation-induced hyperexcitability of trigeminal ganglion (TG) primary neurons and the concomitant mechanical inflammatory hyperalgesia. Rats received complete Freund’s adjuvant (CFA) injections in their whisker pads to induce inflammation. CFA-inflamed rats showed a significantly lower mechanical stimulation-induced escape threshold compared to naïve rats. Systemic butyrate administration restored the mechanical threshold to levels comparable to naïve rats within four days. Four days of butyrate administration significantly decreased the mean increased discharge frequency of TG neurons to both non-noxious and noxious mechanical stimuli in inflamed rats. The increased mean spontaneous discharge of TG neurons in inflamed rats significantly decreased four days after butyrate administration. Collectively, our findings indicate that butyrate reduces inflammatory hyperexcitability in nociceptive primary TG neurons, thereby alleviating inflammatory hyperalgesia. These results suggest that butyrate may serve as a promising therapeutic approach for the prevention of trigeminal inflammatory mechanical hyperalgesia and its clinical manifestations.

## 1. Introduction

Noxious sensory stimuli originating from the territory innervated by small-diameter myelinated Aδ and unmyelinated C trigeminal ganglion (TG) neurons are transmitted via trigeminal afferent fibers to second-order neurons located within the spinal trigeminal nucleus (SpV) in the brainstem [1,2]. The SpV, which serves as a crucial relay point for the transmission of orofacial sensory information, is functionally subdivided into three distinct nuclei: the oralis (SpVo), interpolaris (SpVi), and caudalis (SpVc) nuclei [1,2]. The ability of wide dynamic range (WDR) neurons, located in both primary and secondary neurons, to integrate nociceptive and non-nociceptive inputs [1,2] allows them to encode stimulus intensity in the trigeminal pathway. Specifically, graded nociceptive stimulation of their receptive field elicits a proportional increase in firing frequency. The development of hyperalgesia during persistent pathological conditions, such as tissue inflammation, is mediated by modifications in somatic sensory pathways [3,4]. These modifications involve both peripheral sensitization, characterized by the increased excitability of primary afferent neurons due to inflammation and tissue injury, and central sensitization, resulting from altered signaling within the trigeminal spinal nucleus and higher centers [5]. To explore trigeminal neural signaling pathways associated with pathological pain, researchers have developed CFA models of orofacial inflammation in rats. These models effectively reproduce CFA-induced hyperexcitability of TG neurons, which is closely linked to mechanical stimuli [6,7,8].

The gut microbiota’s influence on biological functions extends beyond digestion to include bidirectional communication with the central nervous system (CNS), as highlighted in recent research. Additionally, studies have linked alterations in gut microbiota composition to the development of various diseases [9]. The influence of gut microbiota on energy metabolism and CNS functions is mediated through the release of humoral factors, including metabolites, neurotransmitters, cytokines, and hormones, and the activation of neural pathways, such as the vagus nerve [9,10,11]. Specifically, short-chain fatty acids (SCFAs), namely acetic, propionic, and butyric acids, are produced via the anaerobic fermentation of dietary fibers by gut microbiota and play a key role in this communication. The interaction of SCFAs with G-protein-coupled receptor 41 (GPR41)/FFAR3 receptors on cell membranes initiates a cascade of intracellular signaling events, ultimately influencing various physiological functions, as suggested by previous studies. Moreover, Nøhr et al. [12] identified GPR41 expression in TG neurons, in addition to its presence in the autonomic nervous system.

The ability of propionic-acid SCFAs to suppress voltage-gated N-type Ca (N-type Cav) channel currents in isolated sympathetic ganglion neurons via GPR41 activation has been established in vitro [13]. Extending this understanding, we have recently shown that systemic SCFAs administration leads to a decrease in nociceptive secondary trigeminal neuron excitability by activating GPR41 signaling, subsequently inhibiting Cav channels at the central terminals of the SpVc [14]. The therapeutic potential of butyrate, a SCFA, in addressing pathological pain, specifically inflammatory and neuropathic pain, is supported by findings from animal models [15,16]. The diverse mechanisms underlying butyrate’s analgesic effects include epigenetic regulation through histone deacetylase (HDAC) inhibition [17], the modulation of nuclear factor-kappa B signaling [18], and direct interaction with G-protein-coupled receptors GPR41 and GPR43 [19]. Although butyrate has demonstrated efficacy in attenuating neuropathic pain, likely through the reduction in inflammatory markers such as cyclooxygenase-2 (Cox-2), inducible nitric oxide synthase, and tumor necrosis factor α [16,20,21], the specific neurophysiological mechanisms by which it modulates nociceptive neuron hyperexcitability remain unclear.

In their study, Ma et al. [16] demonstrated that the phytochemical polyphenol resveratrol significantly attenuated CFA-induced inflammation affecting the temporomandibular joint. Notably, resveratrol also reversed the CFA-induced decrease in butyrate levels and the associated decline in relevant gut bacterial populations. Murakami et al. [22] have shown that the activation of butyrate-GPR41 signaling by *Porphyromonas gingivalis* is a key factor in the pathogenesis of periodontitis in a mouse model, even in the absence of overt periodontal inflammatory pain. Given the accumulating evidence, we hypothesized that butyrate administration would effectively attenuate inflammation-induced hyperexcitability of TG neurons, thereby ameliorating trigeminal hyperalgesia. As this hypothesis remained unexplored, the present study sought to examine, through in vivo experiments, the impact of butyrate administration on the inflammation-induced hyperexcitability of nociceptive primary TG neurons associated with hyperalgesia in rats, utilizing both behavioral and electrophysiological approaches. As a result, the present study provides evidence that butyrate administration attenuates inflammatory hyperexcitability of nociceptive primary TG neurons associated with inflammatory hyperalgesia. These findings support the therapeutic potential of SCFAs, specifically butyrate, for the prevention of trigeminal inflammatory mechanical hyperalgesia, including clinical pain conditions.

## 2. Results

### 2.1. CFA-Induced Orofacial Hyperalgesia

**Figure 1:**
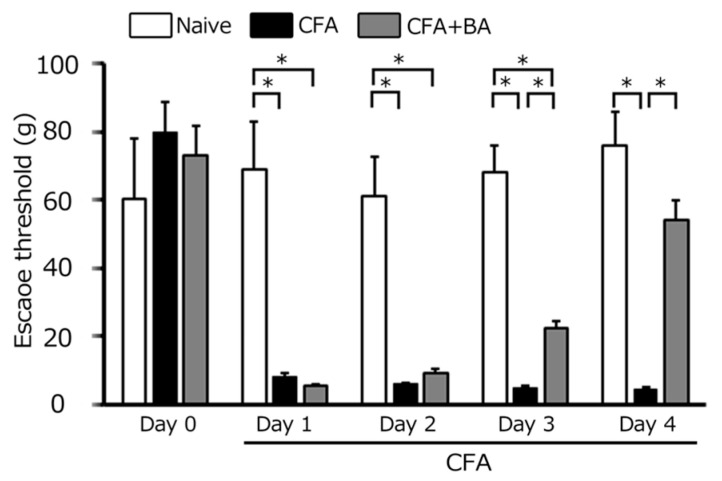
Comparison of changes in escape threshold among naïve, inflamed, and butyrate (BA)-treated inflamed rats. Mechanical stimulation using von Frey filaments was applied to the ipsilateral whisker pad of naïve rats (administered saline), complete Freund’s adjuvant (CFA)-inflamed rats, and CFA-inflamed rats treated with butyric acid (BA) (50 mg/kg, intraperitoneally) to assess mechanical hyperalgesia. Data are presented as mean ± standard error of the mean. Statistical significance was determined by a *p*-value of <0.05 (*), comparing naïve rats (*n* = 5) vs. CFA-inflamed rats (*n* = 6), and CFA-inflamed rats vs. CFA-inflamed rats treated with butyrate (*n* = 6).

The development of inflammatory hyperalgesia following CFA injection into the whisker pad was assessed by quantifying mechanical escape thresholds using von Frey filaments applied to the injected site and orofacial skin. On day 0, no significant difference in escape threshold was observed among the three groups (naïve: 60.2 ± 17.9 g; CFA: 80.0 ± 8.9 g; CFA + BA: 73.3 ± 8.4 g). Consistent with hyperalgesia, Figure 1 demonstrates a significant reduction in mechanical escape threshold in the ipsilateral whisker pad of inflamed rats, decreasing from 76.0 ± 9.8 g in naïve rats to 4.7 ± 0.4 g at day 4 post-CFA injection (*n* = 6, *p* < 0.05; Figure 1). Notably, no significant alteration in mechanical escape threshold was observed in the contralateral whisker pad between the naïve and inflamed groups (60.2 ± 17.9 g vs. 80.0 ± 8.9 g, *n* = 6, NS).

### 2.2. Attenuation of Mechanical Hyperalgesia by Chronic Butyrate Treatment

Daily butyrate administration progressively attenuated the inflammation-induced reduction in the mechanical escape threshold. From day 2 to day 3, a partial recovery was observed, although thresholds remained significantly lower than naïve levels (Figure 1). By day 4, however, butyrate treatment fully reversed the hyperalgesic effect, restoring mechanical escape thresholds in inflamed rats to levels indistinguishable from naïve controls (naïve vs. day 4 inflamed with butyrate: 76.0 ± 17.9 g vs. 54.4 ± 5.7 g, *n* = 6, NS; Figure 1).

### 2.3. Reduction in Inflammatory Edema by Butyrate Treatment: Measurement of Whisker-Pad Thickness

**Figure 2:**
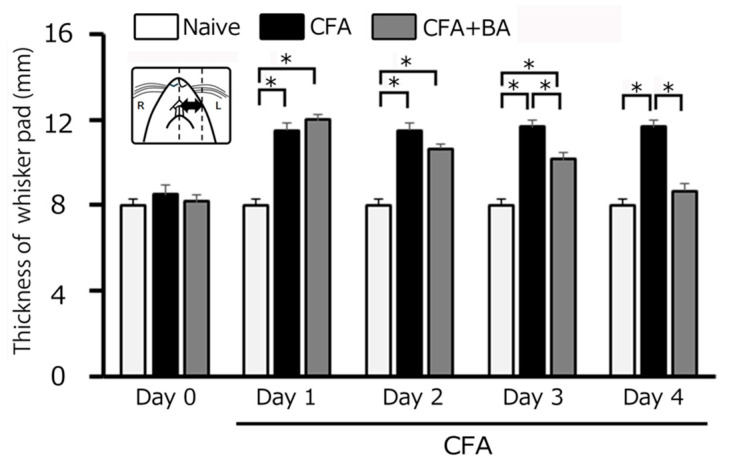
Comparison of changes in complete Freund’s adjuvant (CFA)-induced inflammatory edema among naïve, inflamed, and butyrate (BA)-treated inflamed rats. Each column represents the mean thickness of the edematous area in the whisker pad for three groups of rats. *, *p* < 0.05, comparing naïve rats (*n* = 5) vs. CFA-inflamed rats (*n* = 6), and CFA-inflamed rats vs. CFA-inflamed rats 4 days after butyric acid (BA) (50 mg/kg, intraperitoneal) treatment (*n* = 6). Inset: a schematic diagram illustrating the region used for measuring the CFA-induced orofacial inflammatory edema.

On day 0, there was no significant difference in edema thickness among the three groups (naïve: 8.0 ± 0.3 g; CFA: 8.5 ± 0.4 g; CFA + BA: 8.2 ± 0.3 g). CFA injection induced a significant and sustained increase in whisker-pad edema thickness in inflamed rats compared to naïve controls from day 1 to day 4 (*p* < 0.05). On day 2, the edematous area in inflamed rats reached a mean thickness of 11.5 ± 0.3 mm, compared to 8.0± 0.3 mm in naïve rats (*n* = 6, Figure 2). Importantly, butyrate administration effectively reversed the CFA-induced edema, restoring whisker-pad thickness to control levels by day 4 (naïve vs. inflamed with butyrate: 8.0 ± 0.3 mm vs. 8.7 ± 0.3 mm, *n* = 6, NS; Figure 2).

### 2.4. Inflammation-Induced Alterations in Trigeminal Ganglion (TG) Neuronal Excitability

**Figure 3:**
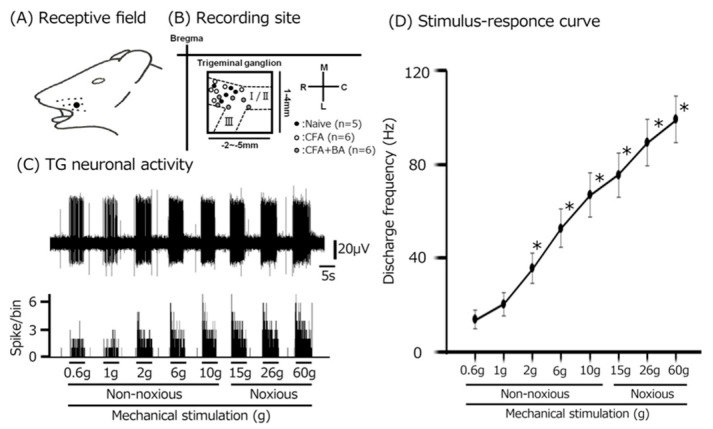
Response properties of trigeminal ganglion (TG) neurons to mechanical stimulation of facial skin. (**A**) Receptive field of the whisker pad on the facial skin (blackened area). (**B**) Distribution of TG neurons responding to non-noxious and noxious mechanical stimulation of the facial skin (*n* = 17) among naïve, CFA-inflamed, and butyrate (BA)-treated CFA-inflamed rats. The inset illustrates an example of TG recording-site identification (2–5 mm posterior to bregma and 1–4 mm lateral to midline). R, rostral; C, caudal; M, medial; L, lateral. Trigeminal nerve branches (I, II, and III). (**C**) Representative examples of TG neuronal activity evoked by non-noxious (2, 4, 6, 8, and 10 g) and noxious (15, 26, and 60 g) mechanical stimulation of the orofacial skin. Upper trace: TG neuronal activity; lower trace: post-stimulus time histogram. (**D**) The stimulus–response relationship for SpVc WDR neurons (*n* = 17). * *p* < 0.05 for comparison of 0.6 g vs. 2 g, 6 g, 10 g, 15 g, 26 g, and 60 g.

To examine the electrophysiological properties of TG neurons involved in whisker-pad mechanosensation, extracellular single-unit recordings were performed across naïve, inflamed, and inflamed + butyrate groups, yielding a total of 17 responsive neurons. As shown in Figure 3A, these neurons exhibited somatic receptive fields within the whisker-pad region, confirming their involvement in processing mechanical stimuli from this area. Recording sites, predominantly located within the maxillary branch of the TG, are depicted in Figure 3B [23]. Importantly, recording site distribution did not differ significantly across the three experimental groups (Figure 3B), ensuring comparability. All analyzed TG neurons displayed a graded increase in firing rate in response to increasing mechanical stimulus intensity, confirming their classification as wide dynamic range (WDR) neurons, a characteristic feature of neurons involved in processing both innocuous and noxious mechanical stimuli (Figure 3C,D) [23].

**Figure 4:**
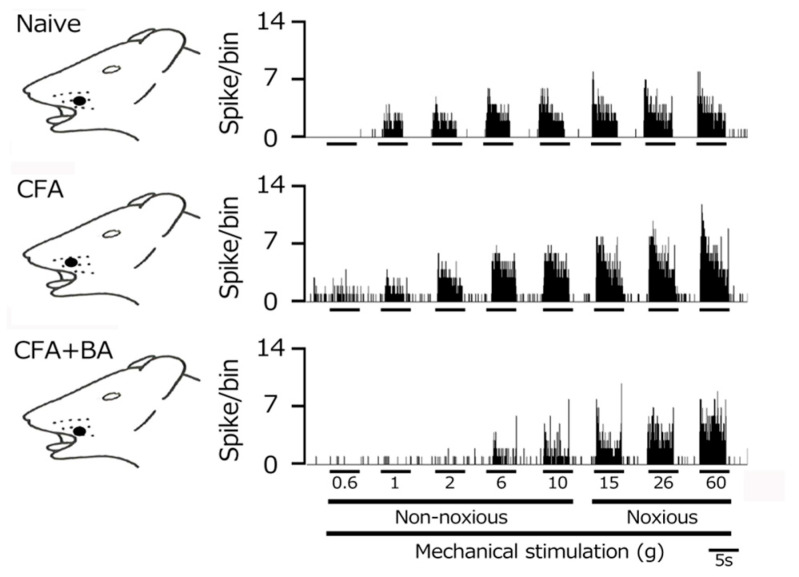
Following orofacial complete Freund’s adjuvant (CFA) inflammation, the hyperexcitability of trigeminal ganglion (TG) neuronal activity was reversed by butyrate (BA) administration. Representative examples of TG neuronal responses to non-noxious and noxious mechanical stimuli are shown for naïve rats, CFA-inflamed rats, and CFA-inflamed rats treated with butyrate (BA) (50 mg/kg, intraperitoneally for four days). The receptive field of the whisker pad on the facial skin is shown (blackened area). Note that the reduced mechanical stimulation threshold and increased spontaneous nerve impulse frequency observed in inflamed rats returned to control levels following four days of butyrate administration.

**Figure 5:**
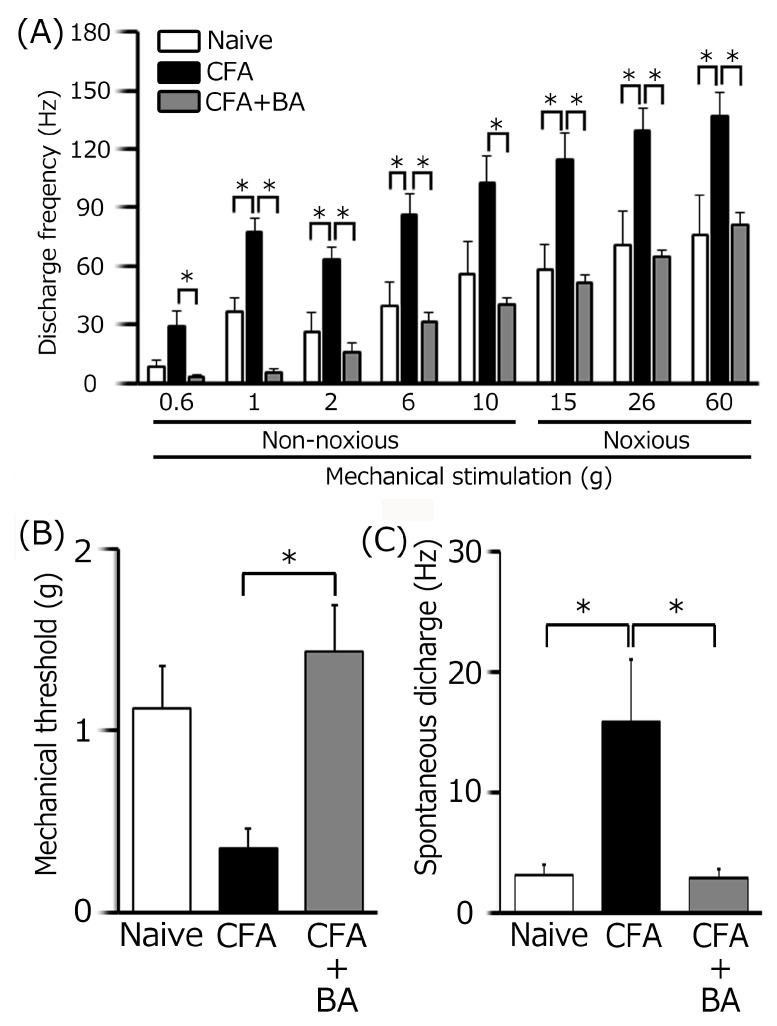
A summary of the effects of chronic butyrate (BA) treatment on elevated trigeminal ganglion (TG) neuronal activity following orofacial inflammation induced by complete Freund’s adjuvant (CFA). (**A**) Average discharge rate of TG neurons in response to non-noxious and noxious mechanical stimulation of the orofacial skin across three groups (*n* = 6). * *p* < 0.05, comparing naïve rats (*n* = 5). vs. CFA-inflamed rats (*n* = 6) and CFA-inflamed rats vs. butyrate-treated CFA-inflamed rats (*n* = 6). (**B**) The mean mechanical threshold of TG neurons across the three groups. * *p* < 0.05, comparing naïve rats vs. CFA-inflamed rats and CFA-inflamed rats vs. butyrate-treated CFA-inflamed rats. (**C**) Spontaneous activity of TG neurons across the three groups. * *p* < 0.05, comparing naïve rats vs. CFA-inflamed rats and CFA-inflamed rats vs. butyrate-treated CFA-inflamed rats.

To confirm the induction of TG neuronal hyperexcitability by CFA, as previously demonstrated [24], we performed extracellular single-unit recordings. In naïve rats, spontaneous discharges were observed in 20% (1/5) of TG neurons (Figure 4 and Figure 5C), with a low mean firing rate of 3.1 ± 0.8 Hz (*n* = 5). In contrast, CFA-induced inflammation resulted in all TG neurons (6/6) exhibiting spontaneous activity (Figure 4 and Figure 5C), indicating increased neuronal excitability. Furthermore, TG neurons in inflamed rats showed significantly enhanced responses to non-noxious mechanical stimulation compared to naïve controls (Figure 4 and Figure 5A) [24]. Mean firing rates elicited by mechanical stimuli (0.6, 1, 2, 6, 10, 15, 26, 60 g) were significantly elevated in inflamed rats (6 g, naïve vs. CFA, 39.1 ± 12.5 Hz vs. 86.0 ± 10.6 Hz; 60 g, 76.1 ± 19. Hz vs. 136.3 ± 12.8 Hz, *p* < 0.05; Figure 5C), and the mean mechanical threshold was significantly decreased to 0.4 ± 0.1 g from 1.1 ± 0.2 g in naïve rats (*n* = 6; Figure 5B), reflecting increased mechanical sensitivity. Consistent with these findings, the mean spontaneous discharge rate was significantly increased in the inflamed group (Figure 5C).

### 2.5. Chronic Butyrate Administration Attenuates Inflammation-Induced Hyperexcitability of TG Neurons

To elucidate the therapeutic effects of chronic butyrate administration on inflammation-induced TG neuronal hyperexcitability, we utilized a behavioral escape threshold assay. Figure 4 illustrates representative TG neuronal discharge rates in response to non-noxious (0.6–10 g) and noxious (15–60 g) mechanical stimulation following four days of butyrate treatment. After this treatment period, TG neuronal discharge frequencies in response to both non-noxious and noxious mechanical stimuli were normalized to control levels (Figure 4), suggesting a reversal of inflammation-induced neuronal sensitization. Butyrate administration also effectively restored the lowered mechanical threshold and augmented spontaneous and evoked firing frequencies observed in inflamed rats to levels comparable to naïve controls. As shown in Figure 5A, mean TG neuronal discharge rates in response to both non-noxious and noxious stimuli were significantly reduced following butyrate administration (6 g, CFA vs. CFA + BA, 86.1 ± 10.6 Hz vs. 31.0 ± 5.4 Hz; 60 g, CFA vs. CFA + BA, 136.3 ± 12.8 Hz vs. 80.7 ± 6.7 Hz, *p* < 0.05), indicating a reduction in neuronal excitability. Similarly, the mean mechanical threshold was significantly reversed to control levels after butyrate treatment (Figure 5B), suggesting a restoration of normal mechanical sensitivity. Furthermore, spontaneous discharge rates of TG neurons were significantly decreased following butyrate administration (CFA vs. CFA + BA, 15.9 ± 5.3 Hz vs. 2.9 ± 0.7 Hz, Figure 5C, *p* < 0.05), suggesting a reduction in neuronal activity. In contrast, chronic vehicle administration had no significant impact on the spontaneous or mechanical stimulation-evoked hyperexcitability of TG neurons in inflamed rats.

## 3. Discussion

### 3.1. Butyrate Administration Mitigates Trigeminal Inflammatory Hyperalgesia

This study aimed to investigate whether, under in vivo conditions involving the systemic administration of the SCFAs, butyrate attenuates inflammation-induced hyperexcitability of TG primary neurons associated with mechanical inflammatory hyperalgesia. In a previous study using a neuropathic pain model, the oral administration of butyrate (100 mg/kg) was reported to significantly suppress nociceptive behavior and COX-2 activity [20]. Given that the bioavailability of orally administered butyrate is approximately 30%, whereas that of intraperitoneally administered butyrate is approximately 80% [25], this study employed intraperitoneal administration of butyrate at a dose of 50 mg/kg in CFA-inflamed animals. In this behavioral study, we identified the following key findings: (i) as previously reported, CFA-inflamed rats exhibited a significantly lower threshold for escape from orofacial mechanical stimulation compared to naïve rats; (ii) chronic butyrate administration for four days reversed this reduced mechanical threshold in inflamed rats to levels comparable to naïve controls; (iii) CFA-inflamed rats showed a significant increase in mean edema thickness compared to naïve rats; (iv) four days of butyrate administration significantly reduced the mean edema thickness in inflamed rats to control levels; and (v) vehicle administration had no significant effect on either the escape threshold or edema thickness in CFA-inflamed rats on day 4. These findings suggest that the systemic administration of butyrate attenuates peripheral sensitization under inflammatory conditions. These behavioral findings are consistent with previous reports of a significant decrease in CFA-induced mechanical hyperalgesia in a rat inflammatory pain model following the application of phytochemicals such as polyphenols and carotenoids [24]. Although the precise mechanism underlying the effects of butyrate on inflammation-induced hyperalgesia remains unclear, several possibilities exist. Butyrate has been shown to decrease prostaglandin E_2_ (PGE_2_) production by inhibiting COX-2 cascades [20]. Taken together, these observations suggest that daily butyrate administration reduces inflammation-induced hyperalgesia in whisker pads through COX-2 suppression, resulting in the inhibition of PGE_2_ production and peripheral sensitization, potentially via the mechanisms described by Syoji et al. [26]. This hypothesis is also supported by evidence that butyrate-based treatment exhibits similar efficacy to that obtained using the potent non-steroidal anti-inflammatory drugs (NSAIDs), such as ketorolac [27]

### 3.2. Potential Mechanisms Underlying the Suppressive Effect of Butyrate on Trigeminal Neuronal Hyperexcitability Associated with Hyperalgesia

The generally accepted mechanism of nociceptive sensory signaling comprises four fundamental processes: (i) the transduction of external stimuli at peripheral terminals; (ii) the generation of action potentials; (iii) the axonal propagation of action potentials; and (iv) synaptic transmission at central terminals, which constitute the presynaptic elements of the initial synapses in central nervous system sensory pathways [28]. Numerous studies have demonstrated that SCFAs interact with GPR41 on cell membranes, thereby influencing physiological processes through diverse intracellular signaling pathways. Furthermore, SCFAs have been shown to modulate the permeability of the blood–brain barrier [29]. To the best of our knowledge, there are currently no reports regarding the modulation of neuronal excitability or action potential generation via GPR41 through voltage-gated, ligand-gated, or transient receptor potential (TRP) channels, with the exception of Cav channels.

In this study, the systemic administration of butyrate reversed the decreased mean mechanical stimulation threshold in inflamed rats. Specifically, both non-noxious and noxious mechanical stimuli-evoked mean discharge frequencies of TG neurons returned to control levels in inflamed rats following butyrate treatment. It is well established that the proinflammatory mediator PGE_2_ binds to G-protein-coupled prostanoid EP receptors, leading to the activation of protein kinase A in nociceptive peripheral terminals during peripheral inflammation [30]. Subsequently, protein kinase A phosphorylates mechanosensitive TRP ankyrin 1 (TRPA1) channels, as well as voltage-gated sodium (Nav) and potassium (Kv) channels. Consequently, the activation threshold of TRPA1 channels is reduced, and membrane excitability increases in the peripheral terminals of TG neurons. These events result in an elevated frequency of nerve impulses being conducted to the presynaptic central terminals of the SpVc. Therefore, our findings suggest that systemic butyrate may modulate inflammation-induced peripheral sensitization and TG neuronal hypersensitivity in peripheral nerve terminals. This aligns with previous in vitro studies demonstrating butyrate’s effects on neuronal activity through the modulation of Cav channels [13].

Cav channels are categorized into two main types: low-voltage activated (T-type) and high-voltage activated (L, P/Q, N, and R-types). To the best of our knowledge, previous studies have demonstrated that SCFAs specifically target N-type Cav channels [13]. Our recent findings demonstrate that the systemic administration of SCFAs suppresses the excitability of nociceptive secondary trigeminal neurons. This suppression occurs through GPR41 signaling-mediated inhibition of Cav channels at the central terminals of the SpVc. These results suggest that SCFAs may serve as effective analgesic agents for the relief of trigeminal nociceptive pain [14]. T-type Cav channels in primary afferent neurons within the pain pathway are essential for maintaining neuronal firing and appear to contribute to neurotransmitter release at afferent terminals in the spinal dorsal horn [31,32]. Increased neuronal excitability leads to the amplification of sensory transmission, resulting in enhanced neuronal excitability, intensified sensory processing, and ultimately, pathological pain perception [33]. Consequently, blocking T-type Cav/Cav3.2 channels mediates analgesia [33]. Gadotti et al. [34] reported that gossypetin, a flavonoid structurally similar to the phytochemical quercetin, suppressed inflammatory and neuropathic pain in peripheral tissues, partially through its action on Cav3.2 channels. Gambeta et al. [33,35] demonstrated that T-type calcium channels are critical regulators of neuronal functions within the trigeminal system and significantly influence trigeminal pain. Therefore, under inflammatory conditions, these findings suggest that chronic butyrate administration may reduce nociceptive TG neuronal excitability via T-type Cav channels expressed in peripheral terminals, leading to the inhibition of Nav and Kv channels in TG neurons. Our previous in vitro study showed that brain-derived neurotrophic factor (BDNF) enhances the excitability of small-diameter nociceptive TG neurons following CFA inflammation through ganglionic BDNF-tyrosine kinase B signaling, which is a potential therapeutic target for trigeminal inflammatory hyperalgesia [8]. Given that BDNF release from TG neurons via paracrine/autocrine mechanisms under inflammatory conditions is dependent on Cav channel activity [8], it can be speculated that butyrate administration, by blocking N- and T-type Cav channels, may decrease the excitability of nociceptive TG neurons in inflammatory states.

**Figure 6:**
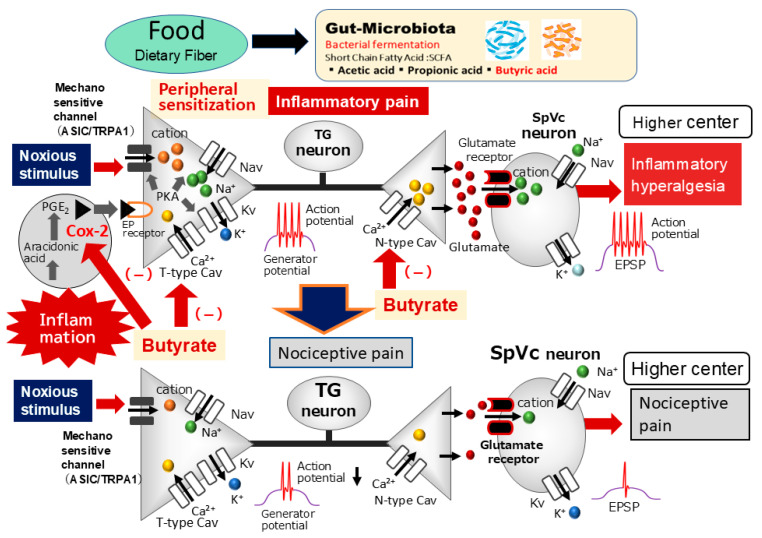
The possible mechanism underlying the systemic administration of butyrate inhibits inflammatory pain. Following peripheral inflammation, inflammatory mediators, such as prostaglandin E2 (PGE_2_), bind to G-protein-coupled E-type prostanoid (EP) receptors and induce the activation of protein kinase (PK) A and PKC in nociceptive peripheral terminals, leading to the phosphorylation of mechanosensitive transient receptor potential ankyrin 1/acid-sensing ion channels (TRPA1, ASIC), voltage-gated Na (Nav), and K (Kv) channels. As a result, the activation threshold for transducer channels, such as the TRP channel family, is reduced, and the membrane excitability of the peripheral terminal increases, resulting in a high frequency of action potentials being conducted to presynaptic central terminals of the trigeminal spinal nucleus caudalis (SpVc). This results in the release of a large amount of glutamate into the synaptic cleft, which binds to upregulated postsynaptic glutamate receptors, augmenting excitatory postsynaptic potentials (EPSPs), causing a barrage of action potentials to be conducted to higher centers of pain pathways, and creating a state of heightened sensitivity, termed peripheral sensitization. The systemic administration of butyrate attenuates the mechanical inflammatory hyperalgesia associated with the hyperexcitability of SpVc neurons via the inhibition of peripheral cyclooxygenase-2 (Cox-2) cascade signaling pathways and the inhibition of N-/T-type voltage-gated Ca (Cav) channels, and this effect restores the SpVc neuronal hyperactivity to control levels.

This study also demonstrated that butyrate reversed the increased mean spontaneous discharge frequency of TG neurons following inflammation. The origin of ongoing activity in central neurons relaying sensory information is of significant clinical interest, as it has been suggested to be a determinant of post-traumatic injury and chronic pain levels [36]. A more recent study showed that the ongoing activity of WDR neurons in the SpVc is driven peripherally, as a microinjection of lidocaine into the trigeminal ganglia significantly decreased this activity [37]. Furthermore, butyrate has been reported to inhibit sodium–potassium–chloride cotransporter activity in the rat colon, leading to increased intracellular chloride ion concentrations [38,39]. Therefore, it can be speculated that this effect in neurons may induce hyperpolarization through increased intracellular chloride ion concentrations and potassium ion efflux, resulting in a reduction in action potential discharge frequency in neurons, including nociceptive neurons. Combined with the present findings, this suggests that butyrate attenuates the increased spontaneous discharge activity of TG neurons innervating the whisker pad, resulting from peripheral and/or trigeminal ganglion sensitization. However, further studies are necessary to fully explore this possibility. Finally, as summarized in Figure 6, we hypothesized the possible mechanisms underlying the suppressive effect of butyrate on trigeminal neuronal hyperexcitability associated with inflammatory hyperalgesia.

### 3.3. Functional Implications of Butyrate’s Attenuating Effect on Trigeminal Inflammatory Hyperalgesia

Regarding trigeminal pain, a previous study demonstrated that butyrate-GPR41 signaling, triggered by *Porphyromonas gingivalis*, plays a critical role in the development of periodontitis without periodontal inflammatory pain in a mouse model [22]. The main findings were as follows: (i) the number of GPR41-immunoreactive TG neurons innervating inflamed periodontal tissue was significantly higher in mice treated with *Porphyromonas gingivalis* compared to CFA-treated and control mice; and (ii) SCFA administration into the gingival tissue of CFA-treated mice restored the reduced head withdrawal threshold [22]. In this study, we found evidence that butyrate administration attenuates the inflammatory hyperexcitability of nociceptive primary TG neurons associated with inflammatory hyperalgesia. Specifically, both inflammation-induced edema in the whisker pad and increased TG neuronal excitability in the inflamed tissues of CFA-inflamed rats were reversed to control levels after four days of butyrate administration. Ma et al. [16] also reported that the phytochemical resveratrol significantly inhibited CFA-induced inflammation in the temporomandibular joint, and reversed the CFA-induced reduction in SCFAs and related gut bacteria. We previously reported that chronic phytochemical administration attenuates CFA-induced mechanical hyperalgesia, and that this effect is due to the suppression of SpVc neuron hyperexcitability via peripheral sensitization through the inhibition of the peripheral Cox-2 signaling cascade [24]. Taken together, these findings suggest that butyrate ameliorates peripheral sensitization in trigeminal inflammatory pain and prevents the further development of central sensitization.

Finally, it is well known that gene expression in nociceptive pathways plays a crucial role in the induction and maintenance of persistent pain, including inflammatory pain [40,41,42]. It has been reported that sodium butyrate is a non-competitive inhibitor of HDAC and selectively inhibits multiple subtypes of Class I and IIa HDACs [43,44]. Given that complete CFA-induced hyperalgesia is inhibited by HDAC IIa inhibitors, previous studies have suggested a key role for HDAC IIa in inflammatory pain [45]. These data indicate that epigenetic regulation within pain pathways participates in the development of persistent pain and analgesic effects. Therefore, it may be tentatively proposed that butyrate-mediated HDAC inhibition is probably responsible for its pain-attenuating effect in trigeminal pathological pain, including persistent inflammatory pain. Given that these findings demonstrate that SCFA administration alleviates CFA-induced inflammatory pain, including trigeminal mechanical hyperalgesia, it can be concluded that SCFAs play a significant role in pain relief. Therefore, further studies are warranted to investigate the potential association between gut microbiota-derived SCFAs and pathological pain conditions, including orthodontic ectopic pain and migraine.

## 4. Materials and Methods

The Animal Use and Care Committee of Azabu University granted approval for all experimental procedures described herein (No. 230120-11). All experiments were conducted in strict accordance with the ethical principles outlined by the International Association for the Study of Pain [46]. Every effort was made to minimize the number of animals used and to alleviate any potential suffering. Furthermore, all researchers performing the experiments were blinded to the experimental conditions to ensure objectivity.

### 4.1. CFA-Induced Orofacial Inflammation and Butyrate Treatment Protocol

To investigate the effects of butyrate on inflammation-induced hyperalgesia, adult male Wistar rats (215–275 g, *n* = 17) were divided into three groups: naïve controls (*n* = 5), rats with CFA-induced inflammation (*n* = 6), and CFA-inflamed rats treated with sodium butyrate (50 mg/kg, i.p.; Sigma-Aldrich, Milano, Italy) (*n* = 6). This dose of butyrate was selected based on previous findings demonstrating its ability to significantly suppress COX-2 activity in vitro [20,21]. Following anesthesia with 3% isoflurane, 0.05 mL of a 1:1 CFA oil/saline suspension was injected into the left facial skin, replicating established protocols [8,26]. To establish baseline conditions, naïve rats received vehicle (0.9% NaCl) injections into the left facial skin and peritoneal cavity. Butyrate, dissolved in 0.9% NaCl, was chronically administered to the treatment group over four days to assess its effects on inflammation. Behavioral experiments were performed daily, immediately prior to butyrate administration. We evaluated the efficacy of butyrate in mitigating peripheral inflammation by measuring the thickness of CFA-induced edema in the whisker-pad region across all experimental groups, as previously detailed [26]. Based on behavioral escape threshold analyses, electrophysiological recordings were conducted on day 4 in the inflamed group to determine neuronal responses. In a subset of experiments, a systemic vehicle (0.9% NaCl) was also administered on day 4 to the inflamed group to isolate the effects of butyrate.

### 4.2. Evaluation of Mechanical Hyperalgesia via Escape Threshold Determination

The mechanical threshold for escape behavior was measured according to established protocols [8,46]. Specifically, from one to four days following CFA or vehicle injection into the facial skin, both ipsilateral and contralateral skin regions were evaluated for mechanical hyperalgesia using a graded series of von Frey filaments (Semmes-Weinstein Monofilaments, North Coast Medical, Morgan, CA, USA). To assess the escape threshold, von Frey mechanical stimuli were applied to the whisker pad in an ascending order of force, with each stimulus application repeated three times. The escape threshold was defined as the minimum force of von Frey stimulation that induced a head withdrawal response in at least one of the three stimulus applications.

### 4.3. Extracellular Single-Unit Recordings of TG Neuronal Activity

Anesthesia was induced in each rat using 3–5% isoflurane followed by an intraperitoneal injection of a mixture containing medetomidine (0.3 mg/kg), midazolam (4.0 mg/kg), and butorphanol (5.0 mg/kg). Anesthesia was subsequently maintained via the intravenous administration of supplemental anesthetic doses (0.25–0.45 mL/kg/h) through a pre-implanted jugular vein cannula, titrated to effect. The adequacy of anesthesia was assessed throughout the recording session by confirming the absence of a reflexive withdrawal response to paw pinching. Rectal temperature was continuously monitored and maintained at 37.0 ± 0.5 °C using a homeothermic blanket (Temperature Controller, 40–90-8D; FHC, Aspen, Tokyo, Japan). All wound margins were regularly infiltrated with 2% lidocaine (Xylocaine) to provide local analgesia. Rats were then secured in a stereotaxic frame (SR-50; Narishige, Tokyo, Japan), and a partial craniotomy was performed, with the center of the opening located 2–5 mm posterior to bregma and 1–4 mm lateral to the midline. To isolate single-unit activity from TG neurons, an enamel-coated tungsten microelectrode (3–5 MΩ) was carefully advanced through the cortex, targeting the TG based on stereotaxic coordinates (2.5–3.5 mm lateral and 2.5–3.5 mm posterior to bregma, 8.1–9.9 mm depth), as previously described [23]. Fine adjustments to electrode position were made in 10 μm steps using a micromanipulator (SM-11 and MO-10; Narishige). Extracellular recordings of single-unit activity were then performed using the tungsten microelectrode (3–5 MΩ), guided by the stereotaxic atlas of Paxinos and Watson [47]. The recorded signals were amplified (DAM80; World Precision Instruments, Sarasota, FL, USA), filtered (0.3–10 kHz) to isolate neuronal spikes, visualized on an oscilloscope (Iwatsu, SS-7672, Tokyo, Japan), and digitized for offline analysis using Power Lab and Chart v.5 software (ADI Instruments, Oxford, UK), following established procedures [23].

### 4.4. Electrophysiological Recording Protocol with Mechanical Stimulation of the Orofacial Region

Extracellular single-unit recordings of trigeminal ganglion (TG) neuronal activity evoked by the mechanical stimulation of the whisker pad were conducted according to the following experimental protocol. To minimize the risk of peripheral mechanoreceptor sensitization, a soft paintbrush was rapidly employed to delineate the approximate boundaries of the receptive field within the left whisker pad [23]. Subsequently, single units exhibiting responses to a series of von Frey filaments, encompassing both non-noxious (1, 2, 4, 6, 8, 10 g) and noxious (15, 26, 60 g) mechanical stimuli, were identified within the left whisker pad [23]. WDR neurons were characterized by their capacity to exhibit a graded increase in firing frequency in relation to escalating mechanical stimulus intensity. Following the identification of nociceptive WDR neurons within the TG responsive to whisker-pad stimulation, mechanical thresholds and receptive field dimensions were meticulously determined [23]. The mechanical receptive field of each neuron was precisely mapped by systematically probing the facial skin with von Frey filaments and subsequently transcribing the delineated area onto a life-sized drawing of the rat on tracing paper [23]. Evoked neuronal discharges induced by mechanical stimulation were quantified by subtracting the baseline spontaneous activity. Spontaneous discharge frequencies were assessed over a 2–5 min interval. Post-stimulus time histograms with 100 ms bin widths were generated for each applied stimulus. Given the established role of WDR neurons within the TG in the pathophysiology of hyperalgesia and referred pain associated with orofacial pain, the present study specifically examined the effects of butyrate on nociceptive WDR neuronal activity, while excluding nociceptive-specific neurons. Post-stimulus time histograms (100 ms bins), mean spontaneous and evoked discharge frequencies, and mean mechanical thresholds of TG neurons were statistically compared across three experimental groups: naïve, CFA-induced inflammation, and CFA-induced inflammation with butyrate treatment.

### 4.5. Statistical Analysis of Behavioral and Electrophysiological Data

Numerical data are presented as the mean ± standard error of the mean. Statistical analyses were performed using one-way repeated-measures analysis of variance to assess differences among multiple groups, with post hoc comparisons conducted using Tukey–Kramer or Dunnett’s tests. For comparisons between two groups, Student’s *t*-tests were used for both behavioral and electrophysiological data (Excel Statcel 4). Statistical significance was defined as a two-tailed *p*-value less than 0.05.

## 5. Conclusions

This study provides evidence that butyrate administration attenuates the inflammatory hyperexcitability of nociceptive primary TG neurons associated with inflammatory hyperalgesia. This attenuation is achieved through the inhibition of peripheral sensitization via the COX-2 signaling pathway and the inhibition of Cav channels. These findings support the therapeutic potential of SCFAs, specifically butyrate, for the prevention of trigeminal inflammatory mechanical hyperalgesia, including clinical pain conditions. Given that only one butyrate concentration was utilized in this study, an additional analysis of concentration dependence is essential to enhance the certainty of the conclusions.

## Data Availability

All data from this study are included in the main body of the article.

## Abbreviations

The following abbreviations are used in this manuscript:

SCFAs	Short-chain fatty acids
TG	Trigeminal ganglion
CFA	Complete Freund’s Adjuvant
SpVc	Trigeminal spinal nucleus caudalis
WDR	Wide dynamic range
CNS	Central nervous system
GPR41	G-protein-coupled receptor 41
Cav	Voltage-gated Ca channel
Nav	Voltage-gated Na channel
Kv	Voltage-gated KJ channel
HDAC	Histone deacetylase
Cox-2	Cyclooxygenase-2
PGE2	Prostaglandin E2
NSAID	Non-steroidal anti-inflammatory drugs
TRPA1	Transient receptor potential ankyrin 1
BDNF	Brain-derived neurotrophic factor
